# Free-Standing Composite Films Based on Thiol-Ene and PEDOT: PSS Layers for Optoelectronic Applications

**DOI:** 10.3390/polym13081299

**Published:** 2021-04-15

**Authors:** Brigita Abakevičienė, Asta Guobienė, Dalius Jucius, Algirdas Lazauskas

**Affiliations:** 1 Institute of Materials Science, Kaunas University of Technology, K. Baršausko 59, LT51423 Kaunas, Lithuania; brigita.abakeviciene@ktu.lt (B.A.); asta.guobiene@ktu.lt (A.G.); algirdas.lazauskas@ktu.edu (A.L.); 2 Department of Physics, Kaunas University of Technology, Studentų str. 50, LT51423 Kaunas, Lithuania

**Keywords:** PETMP-TTT, PEDOT:PSS, composite, layers, free-standing film, mechanical properties

## Abstract

Free-standing composite films were fabricated by combining the plane parallel layers of thiol-ene based on pentaerythritol tetrakis(3-mercaptopropionate)-1,3,5-triallyl-1,3,5-triazine-2,4,6(1H,3H,5H)-trione (PETMP-TTT) UV curable polymer and poly(3,4-ethylenedioxythiophene) poly(styrenesulfonate) (PEDOT:PSS) conductive polymer. A systematic analysis was performed with the focus on mechanical performance of the free-standing composite films. The PEDOT:PSS/PETMP-TTT composite exhibited higher values of adhesion force compared to the free-standing PETMP-TTT film due to hydrophilic nature of the PEDOT:PSS layer. The composite was found to be highly transparent in the range of 380–800 nm. The Young’s modulus and tensile strength of PETMP-TTT were found to be 3.6 ± 0.4 GPa and 19 ± 3 MPa, while for PEDOT:PSS/PETMP-TTT to be 3.5 ± 0.3 GPa and 20 ± 3 MPa, respectively. The sheet resistance values of the PEDOT:PSS layer in the composite film were found to be highly stable after a number of bending iterations with slight increase in sheet resistance from 108 to 118 ± 2 Ω/□. The resultant PEDOT:PSS/PETMP-TTT composite can be further used in optoelectronic applications.

## 1. Introduction

Layered composites consisting of plane parallel layers are important functional materials widely used in different research fields and the industry. These composite materials usually are fabricated by combining layers exhibiting different functional properties which add up to the whole system to meet the practical requirements.

It was demonstrated in [1] that thiol-ene thermoset polymers exhibit shape-memory properties and are capable of recovering their original shape after a temporary deformation when external stimulus is applied. This is, in particular, useful to introduce the specific self-healing mechanism, which facilitates via the bridging of the fractured surfaces into close proximity [2]. These polymerized thiol-ene films also exhibit other attractive properties, such as high optical transparency, homogeneity of the polymer network, negligible oxygen inhibition, low volume shrinkage, flexibility, and toughness [3,4,5].

The poly(3,4-ethylenedioxythiophene) poly(styrene sulfonate) (PEDOT:PSS) conductive polymer has already gained strong position in the market of flexible electronics due to its high electrical conductivity and chemical stability [6]. Thin films of this material also exhibit high optical transmittance in visible spectrum region and thus find numerous applications in different optoelectronic devices, e.g., photovoltaic cells, touch screens, displays, light emitting diodes, etc.

Herein, we report on the design and properties of a new, transparent composite film made of a thiol-ene base and a thin PEDOT:PSS layer. This composite combines self-healing ability of thiol-ene and high electrical conductivity of PEDOT:PSS and could be further used in a number of applications where optical transparency, electrical conductivity, and self-healing properties of the material are required, e.g., in photovoltaics or resistive touch screens. To the best of our knowledge, this is the first attempt to fabricate thiol-ene and PEDOT:PSS composite film, making it stand out in the prevalent research niche of materials for optoelectronic devices.

## 2. Materials and Methods

### 2.1. Materials

Briefly, 1,3,5-triallyl-1,3,5-triazine-2,4,6(1H,3H,5H)-trione (TTT, trifunctional allyl component, ≥97.5%), pentaerythritol tetrakis(3-mercaptopropionate) (PETMP, tetrafunctional thiol component, >95%), 2,2-dimethoxy-2-phenylacetophenone (DMPA, photoinitiator, ≥98.5%), potassium hydroxide (KOH, ≥85.0%), 3,4-ethylenedioxythiophene (EDOT, ≥96.5%), poly(sodium 4-styrenesulfonate) (PSS, >95%), sodium persulfate (Na_2_S_2_O_8_, ≥99%), and ferrous sulfate heptahydrate (FeSO_4_ × 7H_2_O, ≥99%) were purchased from Sigma-Aldrich (St. Louis, MO, USA). All reagents were used without further purification. Ultrapure water with a resistivity higher than 18.2 MΩ/cm at 25 °C was used in all experiments and was obtained from a Direct-Q^®^ 3 UV water purification system (Merck KGaA, Darmstadt, Germany).

### 2.2. Fabrication of the Composite

Photopolymerizable thiol-ene composition was prepared as a mixture of PETMP and TTT with 1:1 stoichiometric ratio of thiol to ene functional groups, containing 1 wt% of DMPA. Details on the preparation procedure have been reported previously [2]. The clear, colorless, viscous mixture of PETMP and TTT was applied on polytetrafluoroethylene (PTFE) plate as a 150 ± 1 µm-thick layer via the Meyer rod coating method. All the samples were UV cured simultaneously at the intensities of 1.64 mW/cm^2^ (254 nm wavelength) and 0.8 mW/cm^2^ (365 nm wavelength) for 5 min. Free-standing PETMP-TTT films were obtained by gently peeling the film from the PTFE plate. The water dispersions of PEDOT:PSS colloids were synthesized by oxidative polymerization of EDOT in the presence of the PSS. Details of the synthesis procedure have been reported previously [7]. The PEDOT:PSS dispersions containing DMSO (50 μL/mL) were spin-coated on the one side of the free-standing PETMP-TTT at 2000 rpm for 30 s. Afterwards, it was dried at room temperature for 24 h (this timing ensures slow evaporation of water molecules, which results in more homogeneous and less defective film) and cured on a hot plate at 125 °C for 2 min. Prior to the spin-coating procedure, the PETMP-TTT side to be coated with PEDOT:PSS was exposed to O_2_ radio frequency (RF) plasma in the camera of the device Plasma-600-T (JSC Kvartz, Kaliningrad, Russia) at 133 Pa pressure (RF = 13.56 MHz, *p* = 0.3 W/cm^2^, t = 30 s) in order to improve wetting and adhesive characteristics of the surface. The resultant two-layer composite was denoted as PEDOT:PSS/PETMP-TTT.

### 2.3. Characterization

Atomic force microscopy (AFM) experiments were carried out at room temperature using a NanoWizardIII atomic force microscope (JPK Instruments, Bruker Nano GmbH, Berlin, Germany), while the data were analyzed using a SurfaceXplorer and JPKSPM Data Processing software (Version spm-4.3.13, JPK Instruments, Bruker Nano GmbH, Berlin, Germany). The AFM images were collected using an ACTA (Applied NanoStructures, Inc., Mountain View, CA, USA) probe (tip shape—pyramidal, radius of curvature (ROC) < 10.0 nm, and cone angle of −20°; silicon cantilever shape—pyramidal, reflex side coating—Al with thickness of 50 ± 5 nm, calibrated spring constant—54.2 N/m, and set point—195.48 nN) operating in contact quantitative imaging mode. The values of the tip radius and tip materials were taken from datasheets provided by the manufacturer of the probes. Adhesion forces were measured by AFM upon detecting the force interaction during approach and retraction of the tip from the sample surface. The 4 μm × 4 μm AFM scan range was divided into a matrix with equal distances such that each point had specific coordinates and the adhesion force, *f*_A_, could be extracted from each position. Adhesion mapping images were constructed by displaying the adhesion force values acquired from the same sample area.

An optical spectrometer Avantes that is composed of a deuterium halogen light source (AvaLight DHc, Avantes, Apeldoorn, The Netherlands) and spectrometer (Avaspec-2048, Avantes, Apeldoorn, The Netherlands) was used to record UV–visible light transmission spectra.

Mechanical properties of the free-standing PETMP-TTT film and PEDOT:PSS/PETMP-TTT composite film were investigated with a custom-build uniaxial tensile tester setup [8]. The later was able to measure sample elongation up to 360 μm. The total strain applied on the sample during tensile testing was 1.4%. The strain rate of tensile experiments was 0.01 mm/s. During tensile testing, the force and displacement of the sample were registered. Samples were cut with an overall length of 40 mm and width of 10 mm for mechanical testing. Mean values of Young’s modulus and yield stress were obtained as an average of five samples tested.

The indentation hardness of the free-standing PETMP-TTT film and PEDOT:PSS/PETMP-TTT composite film was measured using a FischerScope HM 2000S (Fischer, Achern, Germany) with a Vickers indenter. Indentation tests were carried out at different locations on the sample surface. Mean values of indentation modulus and hardness were obtained as an average of five measurements.

Flexibility and stability of the sheet resistance of the PEDOT:PSS/PETMP-TTT were evaluated by the bending test based on collapsing radius method.

Four-point probe testing system (Ossila Ltd., Sheffield, UK) was used for the sheet resistance measurements of the PEDOT:PSS/PETMP-TTT composite before and after bending it multiple times. Probe spacing was 1.27 mm, target current was 50 mA, maximum voltage was 10 V, and voltage increment was 0.010 V. Measurements were performed at room temperature with relative humidity of ~40%.

Raman spectra were recorded using inVia Raman spectrometer (Renishaw, Wotton-under-Edge, UK) equipped with CCD camera and confocal microscope (50× objective). The Raman spectra were excited with 532 nm radiation of semiconductor green laser at 5% output power in order to avoid damage of the sample. The 2400 lines/mm grating was used to record the Raman spectra.

## 3. Results and Discussion

Transparent PETMP-TTT polymer film was used as a base layer of the fabricated composite films because of its scratch-healing at room temperature without external stimulus. The details of the healing process were recently investigated by our group and reported in [2]. Ability of scratch-healing is a highly desirable feature in order to prevent distortion of displayed images and worsening of optical transmittance of protective coatings in touch screens, photovoltaic cells, and similar devices where accidental scratches and cuts tend to accumulate during operation.

**Figure 1:**
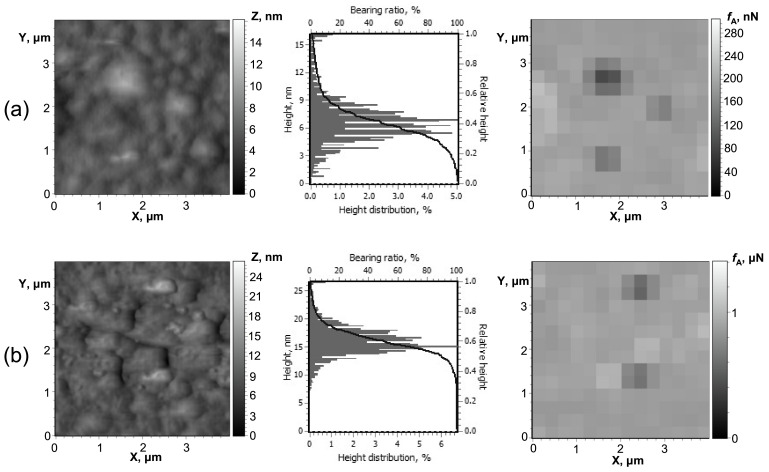
AFM 3D surface topography, normalized height distribution histograms, and bearing ratio curves as well as adhesion mapping images of PETMP-TTT (**a**) and PEDOT:PSS/PETMP-TTT (**b**).

AFM analysis of the free-standing PETMP-TTT film and PEDOT:PSS/PETMP-TTT composite film (Figure 1a,b) was conducted over 4.0 × 4.0 µm^2^ area for quantitative morphological evaluation. The topography of the PETMP-TTT surface exhibits a random distribution of surface features having different angle orientation to each other, without a preferred direction. A mean height of the surface structures (*Z*_mean_) was determined to be 24.2 nm. The root mean square roughness (*R*_q_) was found to be 2.57 nm. The surface peaks dominate over the valleys in the height distribution with a skewness (*R*_sk_) value of 1.13 and exhibits a leptokurtoic distribution of the morphological features with a kurtosis (*R*_ku_) value of 5.84. The *Z*_mean_ and *R*_q_ values for PEDOT:PSS/PETMP-TTT composite were found to be 16.5 and 2.85 nm, respectively. The topography follows similar distribution of surface morphological features with *R*_sk_ and *R*_ku_ values of 0.47 and 5.38, respectively. Adhesion mapping images of the free-standing PETMP-TTT film and PEDOT:PSS/PETMP-TTT composite film are also shown in Figure 1a,b next to topographical information. The adhesion forces observed in adhesion mapping images are in line with the topography of the surfaces. As observed in adhesion mapping images, the surface peaks had lower *f*_A_, while the surface valleys revealed increased tip-sample adhesion. The PEDOT:PSS/PETMP-TTT composite exhibited higher values of adhesion force compared to the free-standing PETMP-TTT film due to hydrophilic nature of the PEDOT:PSS layer, which has a higher tendency to adsorb water molecules from the atmosphere on the surface giving rise to higher van der Waals force [9] resulting in increased adhesion between tip and sample. Low adhesion force for PETMP-TTT is favorable in this composite system as it works as a top protective layer and has lower possibility to attract surface contaminants, which may negatively affect the optical properties of the composite film.

**Figure 2:**
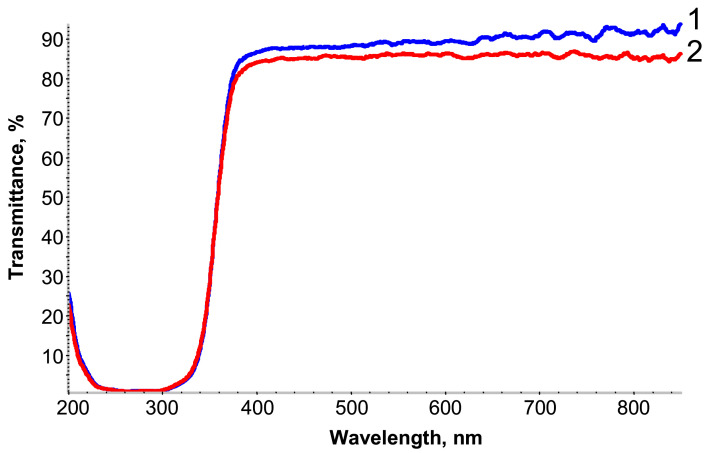
UV–visible transmission spectra of PETMP-TTT (**1**) and PEDOT:PSS/PETMP-TTT (**2**).

The UV–visible transmission spectra of the free-standing PETMP-TTT film and PEDOT:PSS/PETMP-TTT composite film are shown in Figure 2. Only a slight decrease in transmittance of wavelengths in the range of 380–800 nm was observed for PEDOT:PSS/PETMP-TTT composite as compared with the PETMP-TTT film, thus the fabricated composite can be considered highly transparent for visible light.

**Figure 3:**
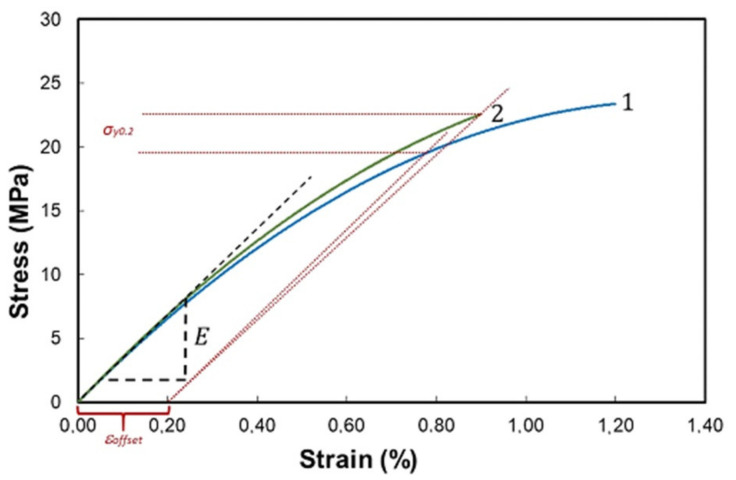
Stress–strain curves of PEDOT:PSS/PETMP-TTT (**1**) and PETMP-TTT (**2**). The characteristic dashed tangential lines represent determination of experimental Young’s modulus (*E*) for the PETMP-TTT free-standing film. The intersection of the dotted lines with the experimental curve at 0.2% strain offset provides the yield stress (*σ_y0.2_*).

Free-standing PETMP-TTT films and PEDOT:PSS/PETMP-TTT composite films were tested in the custom-made tensile setup to determine Young’s modulus, *E* (a slope of the tangent to the stress–strain curve) and yield stress, *σ_y0.2_* (a stress at 0.2% of strain, according to the ε = 0.2% condition). Figure 3 shows the stress–strain curves for the free-standing PETMP-TTT film and PEDOT:PSS/PETMP-TTT composite.

Extrapolating linear elastic parts of the stress–strain curves, the Young’s modulus and tensile strength of the free-standing PETMP-TTT films were determined to be 3.6 ± 0.4 GPa and 19 ± 3 MPa, respectively. The Young’s modulus and tensile strength of the PEDOT:PSS/PETMP-TTT composite films were found to be equal to 3.5 ± 0.3 GPa and 20 ± 3 MPa, respectively. It is evident that differences in Young’s modulus and tensile strength of the tested PETMP-TTT films and PEDOT:PSS/PETMP-TTT composite films are only minor. Moreover, these differences due to the relatively high standard deviation associated with the spread out of the data points over a wide range of values can be considered as statistically insignificant. The obtained results confirm that spin-coating of PEDOT:PSS and subsequent curing at 125 °C do not significantly change the mechanical properties of the PETMP-TTT layer. On the other hand, in our experiments, the PETMP-TTT layer was very thick (150 ± 1 µm) compared to the PEDOT:PSS layer (70 ± 10 nm), standard deviations of measured mechanical properties of PETMP-TTT films were high enough to mask the influence of PEDOT:PSS, and we were unable to obtain the mechanical properties of PEDOT:PSS thin film by subtracting contribution of PEDOT:PSS from the PEDOT:PSS/PETMP-TTT composite. Data provided by other authors show that Young’s modulus for PEDOT:PSS films is smaller compared to the value determined by us for the PETMP-TTT films, whereas tensile strength of PEDOT:PSS is higher. For instance, Lang et al. [10] investigated the tensile properties of PEDOT:PSS thin films in a wide range of relative humidity. The increase in relative humidity from 23% to 55% resulted in decrease in Young’s modulus from 2.8 to 0.9 GPa and decrease in tensile strength from 53.2 to 22.2 MPa. This decrease was explained by the higher amount of water molecules uptake by the hydrophilic and hygroscopic PSS rich shell, which resulted in swelling of the PEDOT:PSS sample and weakened hydrogen bonds. Additionally, Greco et al. [11] investigated properties of the 37.1, 58.3, and 77.1 nm thick PEDOT:PSS thin films and determined Young’s moduli values of 0.81, 1.02, and 1.02 GPa, respectively.

Performed tensile tests have demonstrated that PETMP-TTT films fabricated by our team have sufficient mechanical strength and are suitable to be used as carrying layers of free-standing multifunctional composite films. Young’s modulus of the tested PETMP-TTT films is close to polystyrene but the tensile strength is lower and is close to polyethylene or polypropylene polymers. Softer and more elastic ultrathin layer of PEDOT:PSS will be beneficial to prevent cracks and delamination from the PETMP-TTT substrate thus ensuring integrity of the composite film and stability of its electrical properties.

In another instance, the indentation experiments were carried out in order to determine the indentation hardness of the free-standing PETMP-TTT film and PEDOT:PSS/PETMP-TTT composite. Measurements were performed in accordance with ISO 14577. The indentation hardness for the free-standing PETMP-TTT film and PEDOT:PSS/PETMP-TTT composite was determined to be 4.4 ± 0.7 and 3.2 ± 0.9 N/mm^2^, respectively.

**Figure 4:**
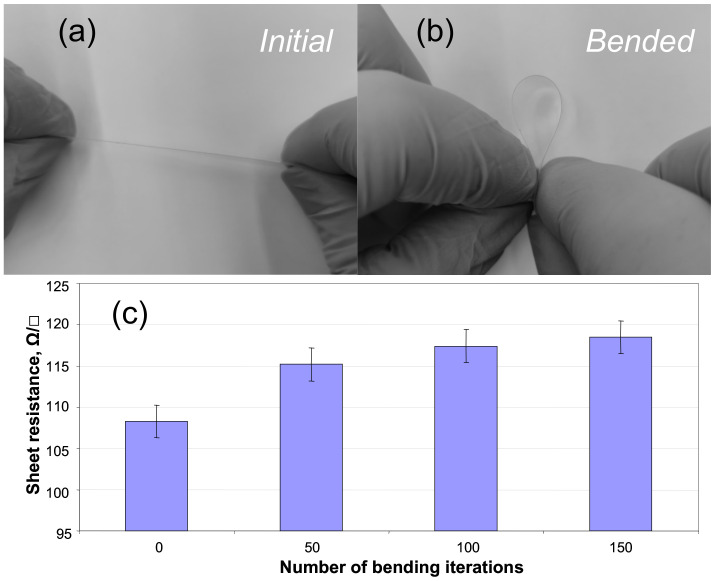
Bending test: free-standing PEDOT:PSS/PETMP-TTT composite film at the initial (**a**) and bended (**b**) position and sheet resistance values of the PEDOT:PSS layer after a number of bending iterations (**c**).

Bending tests have demonstrated high flexibility of the fabricated composite films. Figure 4 shows the free-standing PEDOT:PSS/PETMP-TTT film at the initial and bended position during bending test as well as determined sheet resistance values before and after bending the composite multiple times. The sheet resistance values of the PEDOT:PSS layer were found to be highly stable after a number of bending iterations. The slight increase in sheet resistance from 108 to 118 ± 2 Ω/□ was determined after 150 bending iterations. The exact reasons for recorded change in the sheet resistance are not fully understood. However, they may be related to the rearrangement and interruption of conducting PEDOT chains during cyclic bending.

**Figure 5:**
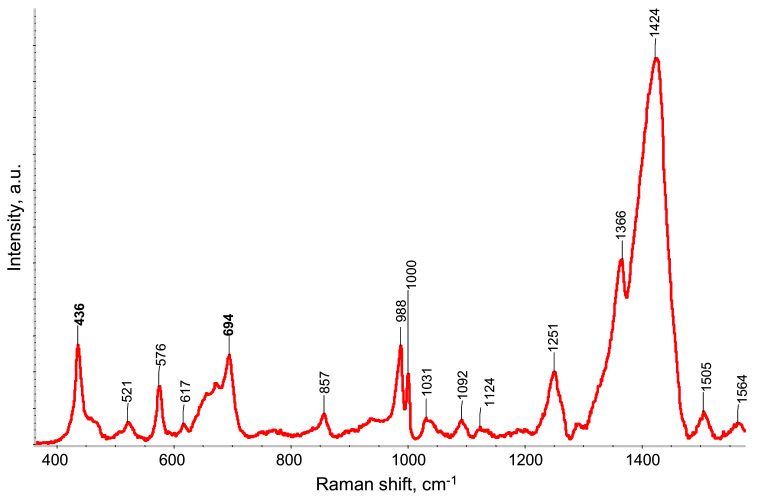
Raman spectrum of free-standing PEDOT:PSS/PETMP-TTT composite film.

Figure 5 shows the Raman spectrum of free-standing PEDOT:PSS/PETMP-TTT film under 532 nm wavelength excitation. The strongest band at 1424 cm^−1^ is assigned to the C_α_= C_β_ symmetric stretching vibration of five-membered thiophene ring (PEDOT), which comes from localized elementary excitations of neutral parts [12,13]. The band at 1505 cm^−1^ is a result of doping, which caused the rearrangement of PEDOT chains, while the band located at 1564 cm^−1^ is attributed to thiophene rings at the end of the PEDOT chains. The band at 1367 cm^−1^ is assigned to the C_β_-C_β_ stretching deformation, while the band at 1251 cm^−1^ is assigned to the C_α_-C_α_ inter-ring stretching vibration of PEDOT [14]. The vibrational modes of PSS component are located at 1124 and 1092 cm^−1^ [15]. The bands associated with the oxyethylene ring deformation-vibrations are located at 988, 857, and 576 cm^−1^ [15,16]. The band located at 694 cm^−1^ is attributed to the C-S-C deformation, while the band at 436 is due to the doping of PEDOT by the SO_3_^−^ ion from PSS units [17].

## 4. Conclusions

Free-standing composite films were fabricated by combining the PETMP-TTT and PEDOT:PSS layers. Quantitative morphological evaluation was performed giving insight on PETMP-TTT and PEDOT:PSS/PETMP-TTT surface topography, roughness, and adhesion force distribution that have been estimated making use of AFM. The fabricated composite was found to be highly transparent for visible light. Mechanical properties (i.e., Young’s modulus, tensile strength, and indentation hardness) of the PEDOT:PSS/PETMP-TTT were tested providing a first characterization of such composite material for its exploitation in optoelectronic applications. Electrical properties and their dependence on the number of bending iterations have been also studied by repeating sheet resistance measurements with a four-point probe technique before and after bending the composite multiple times. It was demonstrated that the electrical properties of PEDOT:PSS/PETMP-TTT composite are highly stable.

## Data Availability

The data of this study will be available from the authors upon request.

## References

[1] Lazauskas A., Grigaliūnas V., Jucius D. (2019). Recovery behavior of microstructured thiol-ene shape-memory film. Coatings.

[2] Lazauskas A., Jucius D., Baltrušaitis V., Gudaitis R., Prosyčevas I., Abakevičienė B., Guobienė A., Andrulevičius M., Grigaliūnas V. (2019). Shape-Memory Assisted Scratch-Healing of Transparent Thiol-Ene Coatings. Materials.

[3] Hoyle C.E., Lowe A.B., Bowman C.N. (2010). Thiol-click chemistry: A multifaceted toolbox for small molecule and polymer synthesis. Chem. Soc. Rev..

[4] Nair D.P., Cramer N.B., Scott T.F., Bowman C.N., Shandas R. (2010). Photopolymerized thiol-ene systems as shape memory polymers. Polymer.

[5] Lowe A.B. (2010). Thiol-ene “click” reactions and recent applications in polymer and materials synthesis. Polym. Chem..

[6] Fan X., Nie W., Tsai H., Wang N., Huang H., Cheng Y., Wen R., Ma L., Yan F., Xia Y. (2019). PEDOT: PSS for flexible and stretchable electronics: Modifications, strategies, and applications. Adv. Sci..

[7] Jucius D., Lazauskas A., Grigaliūnas V., Gudaitis R., Guobienė A., Prosyčevas I., Abakevičienė B., Andrulevičius M. (2019). Structure and Properties of Dual-doped PEDOT: PSS Multilayer Films. Mater. Res..

[8] Arrizabalaga J.H., Simmons A.D., Nollert M.U. (2017). Fabrication of an economical Arduino-based uniaxial tensile tester. J. Chem. Educ..

[9] Bhushan B. (2003). Adhesion and stiction: Mechanisms, measurement techniques, and methods for reduction. J. Vac. Sci. Technol. B Microelectron. Nanometer Struct. Process. Meas. Phenom..

[10] Lang U., Naujoks N., Dual J. (2009). Mechanical characterization of PEDOT: PSS thin films. Synth. Met..

[11] Greco F., Zucca A., Taccola S., Menciassi A., Fujie T., Haniuda H., Takeoka S., Dario P., Mattoli V. (2011). Ultra-thin conductive free-standing PEDOT/PSS nanofilms. Soft Matter.

[12] Ji T., Tan L., Hu X., Dai Y., Chen Y. (2015). A comprehensive study of sulfonated carbon materials as conductive composites for polymer solar cells. Phys. Chem. Chem. Phys..

[13] Xu B., Gopalan S.-A., Gopalan A.-I., Muthuchamy N., Lee K.-P., Lee J.-S., Jiang Y., Lee S.-W., Kim S.-W., Kim J.-S. (2017). Functional solid additive modified PEDOT: PSS as an anode buffer layer for enhanced photovoltaic performance and stability in polymer solar cells. Sci. Rep..

[14] Nguyen T., De Vos S. (2004). An investigation into the effect of chemical and thermal treatments on the structural changes of poly (3,4-ethylenedioxythiophene)/polystyrenesulfonate and consequences on its use on indium tin oxide substrates. Appl. Surf. Sci..

[15] Chang S.H., Chiang C.-H., Kao F.-S., Tien C.-L., Wu C.-G. (2014). Unraveling the enhanced electrical conductivity of PEDOT: PSS thin films for ITO-free organic photovoltaics. IEEE Photon. J..

[16] Park H., Lee S.H., Kim F.S., Choi H.H., Cheong I.W., Kim J.H. (2014). Enhanced thermoelectric properties of PEDOT: PSS nanofilms by a chemical dedoping process. J. Mater. Chem. A.

[17] Kumar S.S., Kurra N., Alshareef H.N. (2016). Enhanced high temperature thermoelectric response of sulphuric acid treated conducting polymer thin films. J. Mater. Chem. C.

